# Compound Danshen dripping pills in treating with coronary heart disease: A protocol for systematic review and meta-analysis

**DOI:** 10.1097/MD.0000000000028927

**Published:** 2022-02-18

**Authors:** Ling Sun, Yan-Na Zhang

**Affiliations:** a Department of Pharmacy, Lianyungang Oriental Hospital, Lianyungang, Jiangsu, China; b Department of Pharmacy, Hefei First People's Hospital, Hefei, Anhui, China.

**Keywords:** compound Danshen dripping pills, coronary heart disease, efficacy, meta

## Abstract

**Background:**

Coronary heart disease (CHD) patients are categorized by occlusion or vascular stenosis leading to myocardial ischemia, hypoxia, and necrosis. In clinical cardiovascular, CHD remains as a leading disease that is primarily prevalent among older people and mid-aged groups. CHD has a drastic impact on their life standard, and is known to have debilitating effects on both mental and physical wellbeing. As a Chinese patent medicine, compound Danshen dripping pills (CDDPs) are commonly administered to treat CHD in China. Despite the common intake of CDDPs, there is a lack of evidence-based clinical practice to inform its efficacy and safety through related systematic reviews. Therefore, the present protocol proposes to conduct a meta-analysis aiming to evaluate the effectivity and safeness of using CDDP for treating CHD patients.

**Methods:**

Randomized controlled trials that have evaluated the efficacy and safety of CDDP for treating CHD patients will be searched in MEDLINE, Cochrane Library, EMBASE, China National Knowledge Infrastructure, and WanFang databases. The search will include all related articles published till January 3, 2022. The extracted data will include information on study design, characteristics of the participants, details on intervention, and outcomes. Cochrane risk of bias tool will be employed to assess the quality of the trials. We will use either a random-effects model or fixed-effects model to pool the data. We will present the results as a risk ratio for dichotomous data and weighted mean difference for continuous data. We will visualize publication bias using funnel plots. Disagreements shall be resolved through discussion.

**Ethics and dissemination:**

Not required.

**OSF registration number:**

10.17605/OSF.IO/HJTP8

## 1 Introduction

Globally, coronary heart disease (CHD) primarily causes cardiovascular-related deaths, with nearly 7.3 million annual fatalities reported, out of which approximately 130,000 have been reported in China.^[^1^–^3^]^ Adopting preventive measures that emphasize healthy habits (physical activity, diet, and not smoking) and following secondary prevention medications reduces the incidence of CHD mortality by at least 47% and reduce 68% of CHD-related primary risk factors.^[^4^–^6^]^ The main characteristics of CHD include coronary atherosclerosis lesions caused by myocardial ischemia, necrosis or hypoxia, occlusion of the lumen, stenosis, and acute inflammation.^[7]^ Recently, 2 types of syndromes were clinically proposed to keep continuously updating the diagnosis and therapy for CHD and formulate therapeutic approaches, including, acute coronary syndrome and chronic myocardial ischemia syndrome.^[^8^,^^9^^]^ Improving myocardial blood supply is vital to clinically treat CHD, which usually involves the intake of antiplatelet agents. Despite advances in modern clinical practice, CHD still has a high prevalence, particularly in developing nations, the disease strains the medical sector, and incurs a substantial financial burden on society, impeding human development.^[^10^,^^11^^]^

In accordance with the fundamental theories of conventional Chinese medicine, CHD's etiology and pathogenesis are linked to blood stasis. Thus, removing blood stasis by promoting blood circulation are primary methods to treat CHD. The compound Danshen dropping pill (CDDP) is primarily composed of *Salvia miltiorrhiza* Bunge, Panax notoginseng, and Borneol. It is effective to improve circulation, removal of stasis, vital energy regulation, and alleviating pain, with particular significance for clinically treating cardiovascular diseases.^[12]^ Improving blood circulation to regulate qi-flowing and eradicate blood stasis to alleviate physical pains are the primary functions of CDDP.^[12]^ It has been reported that CDDP has a considerably healing effect on vascular endothelial functions, relieving angina pectoris and averting restenosis following coronary stenting. However, there is a lack of studies on its effect on coronary artery lesions. Therefore, the present systematic analysis is conducted to evaluate the efficacy and safeness of using CDDP as a treatment for CHD cases.

## 2 Objectives

This research protocol aims to outline a methodical analysis and methodical evaluation to examine the effectiveness and safeness of administering CDDP to treat CHD patients.

## 3 Methods and analysis

The present protocol will be conducted in accordance with the Preferred Reporting Items for Systematic Review and Meta-Analysis Protocols (PRISMA-P) statement.^[13]^ This protocol has been registered in the Open Science Framework (https://osf.io/), registration number: 10.17605/OSF.IO/HJTP8.

## 4 Criteria for considering studies for this review

### 4.1 Types of studies

The proposed systematic review shall consider all randomized controlled trials (RCTs) that examine the effectiveness and safeness of administering CDDP to treat CHD patients.

### 4.2 Types of participants

All participants having CHD will be included, the diagnosis of CHD should adhere to the CHD diagnosis criteria of the clinical diagnosis standards specified by the World Health Organization (WHO), regardless of nationality and gender.^[14]^

### 4.3 Types of interventions

The experimental group will be treated with CDDP, irrespective of the dose, meanwhile, the controls shall be administered other treatment, except CDDP treatment; however, the intake of non-lipid-lowing drugs and non-anticoagulants will be permitted.

### 4.4 Types of outcome measures

Outcome measures include severe adversities related to hemorheology indices, cardiac events, vascular endothelial function indicators, blood lipid parameters, cardiac function indicators, cardiac index, and adverse reactions.

## 5 Search methods for identification of studies

RCTs that assess the effectiveness and safeness of using CDDP to treat CHD patients will be searched in MEDLINE, Cochrane Library, China National Knowledge Infrastructure, EMBASE, and WanFang databases inception to January 3, 2022. The combination of the following search terms will be used: (“compound Danshen dripping pill∗” OR “Fufang Danshen diwan” OR CDDP) AND (“coronary heart disease” OR “CHD” OR “coronary artery disease” OR “CAD”) AND (“randomized controlled trials” OR “randomized controlled studies” OR RCT). Another search will be performed to obtain conference proceedings and the reference lists included in review articles to obtain additional articles. There will be no constraints on language or publication period.

## 6 Data collection and analysis

### 6.1 Selection of studies

The described search strategy shall be employed to source titles and abstracts that could be related to the present review. Subsequently, 2 authors will separately screen the titles and abstracts, and illegible articles will be discarded. All eligible studies and reviews will be retained at this stage.

### 6.2 Data extraction and management

A pair of reviewers will separately evaluate all the obtained abstracts and, if needed, the complete text to ascertain which studies meet the criteria for inclusion. The same authors will perform data extraction independently using standardized data extraction forms. All non-English studies will be translated prior to assessment. In instances where there is more than one publication for a study, we will group the reports and the most updated data set (recent) was considered for analysis. We will highlight all disparities between published versions.

### 6.3 Assessment of risk of bias in included studies

We will assign 2 independent authors to review the systematic quality of the eligible trials using the Cochrane Collaboration’ s tool for bias risk assessment.^[15]^ The tool evaluates the presence of bias in selection by considering the randomization procedures and distribution suppression, performance, and detection of bias by examining the blinding of personnel and outcome assessment, and attrition and bias in reporting by assessing partial and selective data reporting. Each item will be allocated a judgement of high, low, or unclear risk.

### 6.4 Measures of treatment effect

We will express the results as the relative risk with 95% confidence intervals for dichotomous outcomes. Meanwhile, the mean difference will be used to assess treatment effects for continuous scales of measurement. However, if different scales are used, the results will be presented as standardized mean difference (SMD).

### 6.5 Assessment of heterogeneity

A chi-squared test will be adopted on N-1 degrees of freedom to analyze the heterogeneity (*α* = 0.05 denotes statistical significance) and the *I* ^2^ test.^[15]^ *I* ^2^ values of 25%, 50%, and 75% indicate low, medium, and high levels of heterogeneity, respectively.

### 6.6 Assessment of reporting biases

In the case that a large enough number of trials were recognized, funnel plots will be adopted to examine bias in reporting.^[16]^

### 6.7 Sensitivity analysis

We will perform a sensitivity analysis to determine the robustness of our results. Accordingly, we shall omit articles associated with an elevated bias risk from the summary analysis and evaluate the studies by repeating the evaluation to assess how the studies impact the results.

## 7 Discussion

Globally, CHD is a predominant reason behind morbidity and mortality.^[^4^,^^17^^,^^18^^]^ In Western clinical practices, the administration of lifelong aspirin and clopidogrel therapy are regarded as effective ways to reduce fatalities from cardiac problems, myocardial infarction, and strokes.^[19]^ However, there are limitations in dual Western medical antiplatelet therapy, as the abrupt premature stoppage elevates myocardial infarction risk and fatal outcomes.^[^2^,^^9^^]^ Therefore, we will search all related literature without any language constraints to include all relevant trials of CDDP for CHD. The present systematic review and meta-analysis will summarize existing evidence on the effectiveness and safeness of using CDDP to treat CHD patients. The evidence will be a useful reference for clinical practitioners, patients, and health policymakers.

## Author contributions

**Conceptualization:** Ling Sun, Yanna Zhang.

**Data curation:** Ling Sun, Yanna Zhang.

**Formal analysis:** Ling Sun, Yanna Zhang.

**Funding acquisition:** Ling Sun, Yanna Zhang.

**Methodology:** Yanna Zhang.

**Project administration:** Ling Sun.

**Resources:** Ling Sun, Yanna Zhang.

**Software:** Ling Sun.

**Supervision:** Yanna Zhang.

**Validation:** Ling Sun, Yanna Zhang.

**Visualization:** Yanna Zhang.

**Writing – original draft:** Ling Sun, Yanna Zhang.

**Writing – review & editing:** Yanna Zhang.
